# Supplementary material for: Persistence of Campylobacter spp. in Poultry Flocks after Disinfection, Virulence, and Antimicrobial Resistance Traits of Recovered Isolates
Source: Antibiotics (Basel). 2023 May 10;12(5):890. doi: 10.3390/antibiotics12050890 (PMC10215094; doi:10.3390/antibiotics12050890)
Supplement: Supplementary file 1 [file antibiotics-12-00890-s001.zip › antibiotics-2300780-supplementary.pdf]

Supplementary File S1

Date of visit

1. General information

Date of visit

2. Specific information

Poultry: breading hens

Type of rearing: Cage

-Are there any other poultry productions other than those designated on the farm: No

-Are there any other animal productions other than poultry production: No

-Does the farmer have a backyard: No

3. Center infrastructure

Closure: Hard

Front door: Yes

Guard at the door: Yes

Rotoluve: Yes

Device for cleaning and disinfection of vehicles at the entrance of the establishment: Yes

Presence of visitor cloakroom: Yes

4. Building infrastructure

-Presence of airlock: Yes

-Presence of shower: Yes

-Presence of visitor cloakroom: Yes

-Presence of footbaths at the doors: Yes

-Cooling system: Yes

-Presence of sink: Yes

-Ventilation system: static dynamic: Yes

-Grille at the windows: Yes

-Skylight mesh: Yes

-Is there an exchange of equipment from one building to another: No

5. Personnel management

- Staff wear work clothes: Yes

- Work clothes are clean and correct: Yes

-Clothing for visitors: Yes

-Personnel benefits from a medical visit: Yes

Laboratoire d'épidémiologie et de Microbiologie Vétérinaire	Fiche d'enquête	Date d'édition : Mars 2013

Date de visite

1- Renseignements généraux

-Nom du centre : ……………………………………………………………………………………..

- Adresse du centre : …………………………………………………………………………...

- Gouvernorat : …………………………………Délégation : ………………………………..

2- Renseignements spécifiques

- Volailles : Ponte ☐ Chair ☐

- Mode d'élevage : Sol ☐ Cage ☐

- Ya-t-il d'autres productions avicoles autres que celles désigné sur l'exploitation :

Oui ☐ Non ☐

- y-a-t-il d'autres productions animales hors productions avicoles

Oui ☐ Non ☐

- L'éleveur possède t'il une basse cour :

Oui ☐ Non ☐

3- Infrastructure du centre

- Clôture : en dur ☐ grillage ☐ mixte ☐ arbres ☐ absente ☐

- Porte d'entrée : Oui ☐ Non ☐

- Gardien à la porte : Oui ☐ Non ☐

- Rotoluve : Oui ☐ Non ☐

- Fréquence d'entretien du rotoluve : ....

- Produit utilisé : ....

- Dispositif de nettoyage et de désinfection des véhicules à l'entrée de l'établissement

  Oui ☐ Non ☐

- Présence de vestiaires visiteurs: Oui ☐ Non ☐

4- Infrastructure des bâtiments

- Présence de sas : Oui ☐ Non ☐
- Présence de douche : Oui ☐ Non ☐
- Présence de vestiaires visiteurs : Oui ☐ Non ☐
- Présence de pédiluves aux portes : Oui ☐ Non ☐
- Système de refroidissement : Oui ☐ Non ☐
- Présence de lavabo : Oui ☐ Non ☐
- Système d'aération : dynamique statique: Oui ☐ Non ☐
- Si dynamique, système d'aération protégé: Oui ☐ Non ☐
- Grillage aux fenêtres : Oui ☐ Non ☐
- Grillage aux lanterneaux : Oui ☐ Non ☐
- Existe-il un échange de matériel d'un bâtiment à l'autre : Oui ☐ Non ☐

5- Gestion du personnel

- Personnel porte une tenue de travail : Oui ☐ Non ☐
- Tenue de travail est propre et correcte : Oui ☐ Non ☐

Tenue pour les visiteurs : Oui ☐ Non ☐

ᵓersonnel bénéficie d'une visite médicale : Oui ☐ Non ☐

Table S1. PCR primers and conditions used in this study

Species/antibiotics/ Virulence factors	Target Gene	Primer Sequence (5′-3′)	Tm (°C)	Product size (bp)	Reference
Campylobacter spp.	***16S rRNA***	F : GGATGACACTTTTCGGAGC<br>R : CATTGTAGCACGTGTGTC	55	855	[53]
C. jejuni	***mapA***	F :CTATTTTATTTTTGAGTGCTTGTG<br>R :GCTTTATTTGCCATTTGTTTTATTA	55	589	[61]
C. coli	***cdtA***	F : ATTGCCAAGGCTAAAATCTC<br>R : GATAAAGTCTCCAAAACTGC	52	370	[62]
**Tetracycline**	***tet*(O)**	F: GCGTTTTGTTTATGTGCG<br>R: ATGGACAACCCGACAGAAG	54	559	[63]
**Multidrug CmeABC efflux system**	***cmeB***	F: AGGCGGTTTTGAAATGTATGTT<br>R: TGTGCCGCTGGGAAAAG	52	444	[64]
**Ampicillin/Amoxicillin**	***bla*$_{OXA-61}$**	F: AGAGTATAATACAAGCG<br>R: TAGTGAGTTGTCAAGCC	52	372	[65]
**Gentamicin**	***aphA-3***	F: TGCGTAAAAGATACGGAAG<br>R: CAATCAGGCTTGATCCCC	52	701	[65]
**Erythromycin**	***23S rRNA***	23S rRNA: TTAGCTAATGTTGCCCGTACCG<br>ERY2075: TAGTAAAGGTCCACGGGGTCGC<br>ERY2074: AGTAAAGGTCCACGGGGTCTGG	50	485	[59]
**Ciprofloxacin /Nalidixic acid**	***gyrA-Cj***	gryA1: TTTTTAGCAAAGATTCTGAT<br>gyrA5: AAAGCATCATAAACTGCAA<br>gyrA4: CAAAGCATCATAAACTGCAG	52	265<br>368	[58]
	gyrA-Cc	gyrA3: TATGAGCGTTATTATCGGTC<br>gyrA8: TAAGGCATCGTAAACAGCCA<br>gyrA4: GTCCATCTACAAGCTCGTTA	55	192<br>505	[57]
FlaA protein (motility)	***flaA***	F:AATAAAAATGCTCATAAAAACAGGTG<br>R :TACCGAACCAATGTCTGCTCTGATT	55	855	[66]
CadF, outer membrane protein (adhesion)	***cadF***	F : TTGAAGGTAATTTAGATATG<br>R : CTAATACCTAAAGTTGAAAC	54	400	[69]
Cytolethal distending toxin subunit (CDT toxin production)	***cdtA***	F : CCTTGTGATGCAAGCAATC<br>R : ACACTCCATTTGCTTTCTG	52	370	[68]
Cytolethal distending toxin subunit B (CDT toxin production)	***cdtB***	F : CAGAAAGCAAATGGAGTGTT<br>R : AGCTAAAAGCGGTGGAGTAT	51	620	[66]
Cytolethal distending toxin subunit C (CDT toxin production)	***cdtC***	F : CGATGAGTTAAAACAAAAAGATA<br>R : TTGGCATTATAGAAAATACAGTT	47	182	[66]

Type IV secretion system (invasion)	***virB11***	F : TCTTGTGAGTTGCCTTACCCCTTTT<br>R : CCTGCGTGTCCTGTGTTATTTACCC	48	494	[66]
1,3 galactosyltransferases involved in lipopolysaccharide production (GBS)	***wlaN***<br><br>***CgtB***	F : TTAAGAGCAAGATATGAAGGTG<br>R : CCATTTGAATTGATATTTTTG<br>F :TAAGAGCAAGATATGAAGGTG<br>R :GCACATAGAGAACGCTACAA	46<br><br>49	672<br><br>561	[53]
CeuE, lipoproteininvolved in iron acquisition	***ceuE- Cj***<br><br>***ceuE-Cc***	F :CCTGCTACGGTGAAAGTTTTGC<br>R :GATCTTTTTGTTTTGTGCTGC<br>F :ATGAAAAAATA TTTAGTTTTTGCA<br>R :ATTTTATTATTTG TAGCAGCG	48<br><br>48	793<br><br>462	[69]
